# Time-of-day effects on blood lactate levels following the Wingate anaerobic test in trained women athletes

**DOI:** 10.1186/s13102-026-01665-1

**Published:** 2026-03-26

**Authors:** Yakup Köse, Mehmet Ulaş, Hakan Büyükçelebi, Ulaş Sadık Bardakçı

**Affiliations:** 1 Faculty of Sport Science, Burdur Mehmet Akif Ersoy University, Burdur, Türkiye; 2 Faculty of Sport Science, İnönü University, Malatya, Türkiye; 3 Institute of Educational Sciences, Burdur Mehmet Akif Ersoy University, Burdur, Türkiye

**Keywords:** Anaerobic power, Blood lactate level, Time of day, Wingate, Ratings of perceived exertion

## Abstract

This study aimed to examine the effect of time of day on anaerobic performance, blood lactate responses, and perceived exertion following the Wingate Anaerobic Test in trained women athletes engaged in regular resistance training. Twenty-one participants completed the test during three separate sessions conducted in the morning, afternoon, and evening. Ratings of perceived exertion were recorded immediately after exercise, while blood lactate concentrations were measured at baseline, 3 min post-exercise, and 33 min post-exercise to assess peak and recovery responses. The results demonstrated a significant effect of time of day on anaerobic performance, with peak and average power output significantly higher in the evening than in the morning (*p* < 0.01), and average power output also higher in the afternoon than in the morning. In contrast, fatigue index, blood lactate concentrations, and perceived exertion did not differ significantly across the different time points. These findings indicate that anaerobic power output varies with time of day, whereas fatigue index, blood lactate concentrations, and perceived exertion remain unaffected.

## Introduction

Recent research in chronobiology has concentrated on the impact of the time of day on athletes' performance, particularly regarding physical capabilities. Understanding the mechanisms underlying circadian rhythms' influence on anaerobic performance has consistently presented a significant challenge [14]. Recent studies indicate a substantial correlation between circadian rhythms and anaerobic performance [17, 53]. Human circadian rhythms create an internal environment that varies consistently and predictably throughout the day [39]. As a result, individuals exhibit variations in arousal [49], strength [5], cardiorespiratory capacity [46], and other performance-related factors between morning and evening. Motor performance changes associated with circadian rhythms have primarily been characterized by long- and medium-term efforts, which are predominantly influenced by cardiorespiratory endurance [6, 42]. Circadian rhythms may also impact short-term competitive events that rely on maximal physical strength and power output [52, 58]. The relationship between peak anaerobic performance and the circadian rhythm of temperature remains a topic of debate [14].

The 30-s Wingate Anaerobic Test (WAnT 30) is widely used to assess anaerobic power characteristics and has demonstrated validity and reliability [7]. Biological rhythm is a crucial factor influencing short-term maximal performance, as exemplified by the Wingate test and brief efforts such as maximal voluntary contraction [56]. The underlying reasons for these diurnal oscillations remain unclear [20], and additional factors, including testing protocols and athletes' training experience, contribute to the variability in results. The impact of circadian rhythm on sports performance is significant for athletes and coaches, affecting both immediate and long-term achievements of individuals or teams [18]. Determining the impact of the time of day on short-term maximal performance is essential for athletes, coaches, and researchers aiming to achieve peak performance [1]. Throughout the day, various components of physical performance—such as muscle strength and cardiorespiratory endurance—fluctuate, with higher performance levels observed during midday and early evening and lower levels during late night and early morning hours [34, 42, 48, 57].

In addition to performance-related outcomes, recent attention has also been directed toward understanding how time of day influences physiological responses such as blood lactate concentration. However, research on circadian rhythms and time-of-day variation in lactate responses is limited, and existing studies findings are contradictory. Some studies suggest that post-exercise blood lactate concentrations may vary at different times of the day and reflect underlying changes in glycolytic flux, metabolic clearance, or hormonal regulation [9, 28]. Conversely, some studies indicate that blood lactate concentrations do not vary depending on the time of day [33, 51]. Yet, the direction and magnitude of these fluctuations remain inconsistent, and methodological differences across studies make it difficult to draw definitive conclusions. Further research is needed to clarify how circadian timing may influence lactate production and clearance following high-intensity efforts.

Nonetheless, the majority of studies in this field have focused almost exclusively on male participants, leading to a notable gender gap in the literature [18, 26, 31, 33, 52, 53]. Sex-specific hormonal profiles, neuromuscular recruitment patterns, and fatigue-related physiological mechanisms may influence the magnitude and timing of circadian modulation in anaerobic performance and post-exercise metabolic responses, including blood lactate dynamics [8, 60]. However, these sex-related differences remain insufficiently explored in trained women athletes, limiting the generalizability of existing findings and underscoring the need for female-specific research. To address these gaps, the present study investigated the effects of time of day on anaerobic performance and blood lactate responses in trained women athletes. Under standardized laboratory conditions, participants performed the Wingate Anaerobic Performance Test at three distinct time points (morning, afternoon, and evening). By focusing on a homogeneous group of women athletes with consistent training backgrounds, this study aims to contribute to a more inclusive and comprehensive understanding of circadian influences on anaerobic performance and blood lactate responses.

## Materials and methods

### Participants

Participants were recruited from women students enrolled in the Faculty of Sport Sciences who met the study inclusion criteria. After applying the inclusion criteria for women who had been engaging in regular strength training at least 3 times per week for the past 12 months, 21 trained women were included in the study. The mean age of the participants was 21.33 ± 1.88 years, the mean height was 166.52 ± 7.07 cm, the mean body weight was 61.88 ± 9.17 kg, and the body fat percentage was 24.09 ± 3.55 (see Table 1). The inclusion criteria for the study were as follows: (1) participants were women aged 20 to 25 years; (2) they had been performing strength training at least three days per week for the past 12 months; and (3) they had no injuries to skeletal muscles, joints, or tendons in the past two years. Participants who did not meet these criteria or were unable to comply with the pre-test requirements were not eligible for inclusion. Participants were instructed to abstain from physical activity, alcohol, and caffeine-containing products, including caffeinated beverages and dietary supplements, for 24 h before each testing session. In addition, participants were asked to record all food and beverage intake and to maintain a consistent diet during the 24 h preceding subsequent test sessions.

**Table 1:** Demographic characteristics of the participants

	Mean/Std
Age	21.33 ± 1.88
Height (cm)	166.52 ± 7.07
Body weight (kg)	61.88 ± 9.17
Fat Percentage (%)	24.09 ± 3.55

### Experimental design

**Fig. 1:**
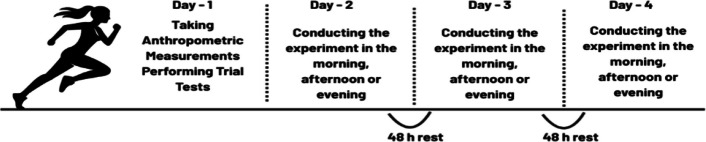
Experimental Design

This study employed a randomized, balanced crossover design to examine time-of-day effects on blood lactate responses following the Wingate Anaerobic Performance Test (30 s). Each participant attended the laboratory on four separate occasions. During the first visit, participants underwent body composition assessments. Subsequently, each participant completed three experimental sessions scheduled in the morning (08:00–09:00), afternoon (13:00–14:00), and evening (18:00–19:00). The order of the testing sessions was randomized and counterbalanced so that all participants completed each time-of-day condition an equal number of times as the first, second, and third sessions. The randomization sequence for the test conditions was generated electronically using the website https://www.randomizer.org and concealed until the start of data collection. A 48-h recovery period was provided between sessions to minimize carryover effects (see Fig. 1).

**Fig. 2:**
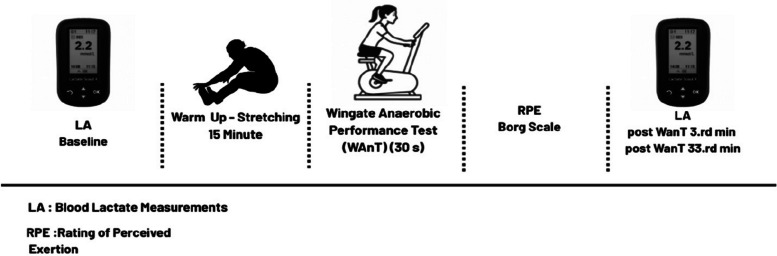
Experimental protocol and measurement sequence during each testing session

During each testing session, participants reported to the laboratory at the assigned time. Basal blood lactate levels were obtained from the earlobe using a hypodermic pen needle before testing. A standardized warm-up protocol was then applied, including adjustment of the bicycle ergometer seat height, to ensure adequate preparation for the Wingate Anaerobic Performance Test. Participants subsequently completed the 30-s Wingate test. Immediately after the test, perceived exertion was assessed using the Borg Rating of Perceived Exertion scale (RPE) (6–20). Blood lactate samples were collected again at the 3rd and 33rd minutes following test completion using the same sampling method. During the post-test period, participants remained seated in a laboratory environment maintained at 23 °C and performed passive recovery. Environmental conditions were monitored using a VZN Medical Device Hydrothermograph, and all rest intervals were timed and supervised by the researchers using a stopwatch to ensure standardization across sessions. The overall experimental protocol and measurement timeline are illustrated in Fig. 2.

### Data collection tools

#### Body composition measurements

Each participant's height was measured to the nearest 0.1 cm using a stadiometer (Holtain Ltd., Crymych, UK). During the measurement, participants stood barefoot in an upright posture with their heads positioned according to the Frankfurt horizontal plane to ensure standardization. Body composition was analyzed using a multi-frequency bioelectrical impedance analyzer (InBody 270, Biospace Co., Ltd., Seoul, Korea), which has demonstrated acceptable agreement with reference methods such as dual-energy X-ray absorptiometry in healthy populations. This device utilizes ten impedance measurements at 20 and 100 kHz across five distinct body segments (right arm, left arm, trunk, right leg, and left leg). The assessment lasted approximately 15 s. During this time, participants stood barefoot on the analyser’s electrodes and held the hand grips, following the manufacturer’s standardised protocol [38].

### Wingate anaerobic performance test

The Wingate anaerobic performance test was conducted using a friction-loaded bicycle ergometer (Monark Ergomedic 894E, Monark, Stockholm, Sweden) connected to a microcomputer. A standard warm-up protocol was implemented before the test to prepare the muscles and enhance the validity of the results. Participants pedaled on the ergometer for 5 min at a cadence of 70 revolutions per minute (rpm) against a constant load of 60 W. During the warm-up, participants performed two 5-s unloaded sprints in the 2nd and 3rd minutes to activate the anaerobic energy systems and stimulate the neuromuscular system. Participants rested for a few minutes following the warm-up before proceeding to the primary test. The Wingate anaerobic test was performed in accordance with the protocol outlined in [7]. Participants pedaled with maximum effort for 30 s against a resistance load of 7.5% of their body mass (0.075 kg ⁻^1^). The test was initiated with a "rolling start" at minimum resistance, with the weight basket supported, and participants began pedaling at 50 rpm. When the pedaling speed reached 130 rpm, the weight basket automatically dropped. From this moment on, participants were asked to pedal with maximum effort for the 30-s test period. Verbal motivation was provided throughout the test, encouraging the maintenance of maximum effort. The data obtained during the test were transferred to the computer via the Monark Anaerobic Test Software (Version 3.3.0.0). With the software, data from each moment of the test were recorded and analyzed. From these data, parameters such as Peak Power (W), Relative Peak Power (W/kg), Average Power (W), Relative Average Power (W/kg), Minimum Power (Min W), and Fatigue Index (FI = [(Peak Power − Min power)/Peak Power] × 100) were obtained.

### Blood lactate level measurements

In the present study, blood lactate concentrations were determined using the Lactate Scout 4 (EKF Diagnostics, Cardiff, United Kingdom), a portable analyzer that operates on electrochemical detection principles. The device requires only 0.5 µL of capillary blood and can measure lactate concentrations within a range of 0.5 to 25 mmol/L. It provides high precision, with a measurement error margin limited to ± 0.2 mmol/L. Each measurement is completed in less than 10 s, enabling fast and efficient data acquisition. Moreover, the device is equipped with automatic calibration and integrated quality control features through test strips, ensuring the validity and reliability of the recorded data.

Lactate measurements were obtained from capillary blood samples drawn from the participants’ earlobes at three specific time points: (1) prior to the test to determine baseline levels, (2) at the 3rd min post-exercise to capture peak lactate concentrations, and (3) at the 33rd min following the test to evaluate the lactate recovery response. These sampling points were strategically selected to assess the acute metabolic response to maximal anaerobic effort and the subsequent clearance dynamics during the recovery phase.

### Rating of perceived exertion

RPE Scale was used to assess participants’ subjective effort following high-intensity anaerobic exercise. This psychophysiological scale, which ranges from 6 to 20, allows individuals to rate the perceived physical demand of exercise and has demonstrated strong criterion-related validity with physiological markers of exercise intensity in healthy individuals [11, 15]. Incorporating the RPE scale into the methodology enabled quantification of participants’ internal responses to the Wingate anaerobic test, which was administered at three different times of day. When considered alongside blood lactate concentrations, these perceptual ratings offer a more comprehensive understanding of physiological and psychological responses to supramaximal exertion at various time points.

### Statistical analysis

The minimum required sample size for the present study was determined using G*Power software (University of Düsseldorf, Düsseldorf, Germany) [22]. An a priori power analysis using F tests was conducted based on the study design, which included a repeated-measures ANOVA with three time points as within-subject factors. The parameters specified for the analysis included a significance level (α) of 0.05, a moderate effect size (f) of 0.30, three repeated measurements, and a desired statistical power (1 − β) of 0.80. Although previous studies in similar research contexts have reported effect sizes of 0.30 or greater [21, 31, 32, 62], a conservative approach was adopted, selecting an effect size of f = 0.30. Based on these parameters, the analysis indicated that a minimum sample size of 20 participants was required, corresponding to an actual statistical power of 81.2%.

All statistical analyses were performed using JASP software (version 0.19.3) [61]. Differences between time-of-day measurements were examined using a single-factor repeated-measures ANOVA. In addition, Bayesian repeated-measures ANOVA was conducted to evaluate the probabilistic strength of the observed effects, following the reporting recommendations for Bayesian analyses [59]. Data normality was assessed using the Shapiro–Wilk test. The assumption of sphericity was evaluated using Mauchly’s test; when violations were detected, the Greenhouse–Geisser correction was applied. Pairwise comparisons were performed for variables showing significant main effects, with Bonferroni adjustments used to control for Type I error.

The magnitude of changes between measurement times was expressed as Cohen’s d, together with 95% confidence intervals. Effect sizes were interpreted according to the thresholds proposed by [30]: < 0.2 negligible, 0.2–0.6 small, > 0.6–1.2 moderate, > 1.2–2.0 large, and > 2.0 very large. For Bayesian analyses, both the Bayes factor (BF₁₀) and the inclusion Bayes factor (BF_incl) were reported. Statistical significance was set at *p* < 0.05.

## Results

### Wingate anaerobic performance test results

Wingate anaerobic performance outcomes demonstrated a clear time-of-day effect on power-related variables, whereas the FI remained unaffected by the timing of measurement. Analysis revealed a significant effect of time of day for both relative peak power (PPO, W·kg⁻^1^) [*F*(2, 40) = 12.050, *p* = 0.001, η^2^ₚ = 0.376] and relative average power (APO, W·kg⁻^1^) [*F*(2, 40) = 18.230, *p* = 0.001, η^2^ₚ = 0.477]. In contrast, no significant time-of-day effect was observed for the FI [*F*(2, 40) = 2.696, *p* = 0.085, η^2^ₚ = 0.119] (see Table 2). Bayesian analyses supported these findings, providing strong evidence for including the time factor in the models for relative peak power (BF_incl = 757.410) and relative average power (BF_incl = 16,774.357), while offering no meaningful evidence for a time effect on the FI (BF_incl = 0.937).

**Table 2:** Fatigue Index (FI)

TİME	Mean/Std	Fatigue Index (FI)				
		F	P	n2	BF10	BF ıncl
Morning Test	61.00 ± 9.21	2.696	0.085	0.119	1.000	0.937
Afternoon Test	56.18 ± 7.63					
Evening Test	57.54 ± 9.44					

**Fig. 3:**
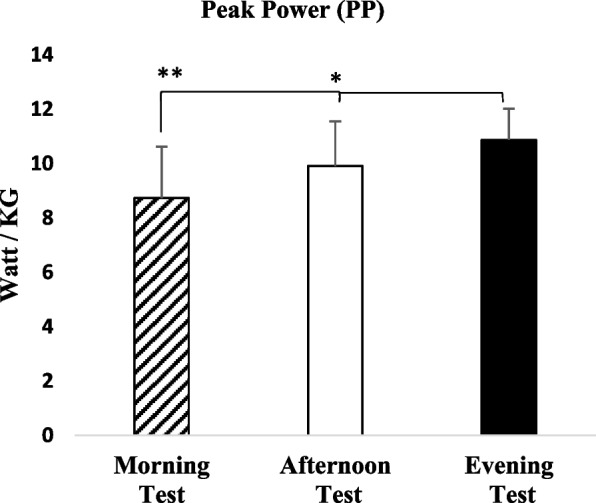
Peak Power (W·kg⁻^1^) measured in the morning, afternoon, and evening during the Wingate anaerobic test. Error bars represent standard deviation.*: *p* < 0.05: Statistically significant difference between afternoon and evening in favor of evening. **: *p* < 0.05: Statistically significant difference between morning and evening in favor of evening

Post hoc comparisons for relative peak power indicated significantly higher values in the evening compared with both the morning (*p* = 0.001; 95% CI = 1.01–3.25; Cohen’s *d* = 1.34; BF₁₀ = 369.418) and afternoon (*p* = 0.044; 95% CI = 0.02–1.90; Cohen’s *d* = 0.60; BF₁₀ = 3.679). No statistically significant difference was observed between morning and afternoon measurements (*p* = 0.093; 95% CI = − 0.14–2.49; Cohen’s *d* = 0.73; BF₁₀ = 1.988). Consistent with these comparisons, relative peak power increased by 10.46% in the afternoon and by 18.39% in the evening compared with morning measurements, with an additional 8.87% increase observed between the evening and afternoon sessions (see Fig. 3).

**Fig. 4:**
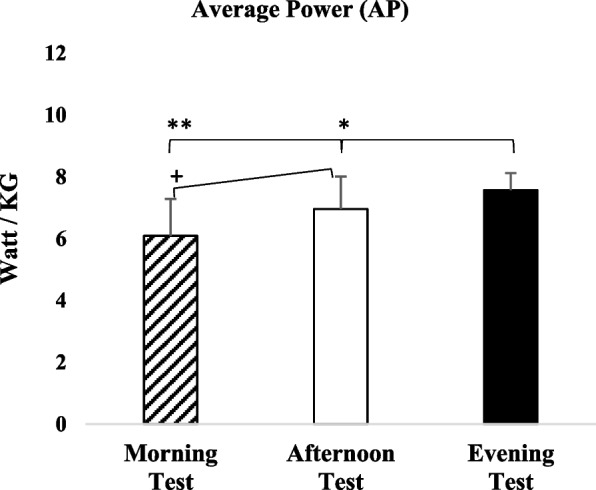
Average Power (W·kg⁻^1^) measured in the morning, afternoon, and evening during the Wingate anaerobic test. Error bars represent standard deviation. *: *p* < 0.05: Statistically significant difference between afternoon and evening in favor of evening. **: *p* < 0.05: Statistically significant difference between morning and evening in favor of evening. + : *p* < 0.05: Statistically significant difference between morning and afternoon in favor of afternoon

A similar pattern was observed for relative average power. Evening measurements were significantly higher than both morning (*p* = 0.001; 95% CI = 0.76–2.20; Cohen’s *d* = 1.51; BF₁₀ = 816.372) and afternoon values (*p* = 0.019; 95% CI = 0.08–1.13; Cohen’s *d* = 0.62; BF₁₀ = 7.353). In addition, afternoon measurements were significantly greater than morning values (*p* = 0.009; 95% CI = 0.19–1.53; Cohen’s *d* = 0.88; BF₁₀ = 14.394). These differences were accompanied by increases of 12.50% in the afternoon and 19.55% in the evening relative to the morning, as well as an 8.06% increase favoring the evening compared with the afternoon (see Fig. 4).

### Blood lactate level results

**Fig. 5:**
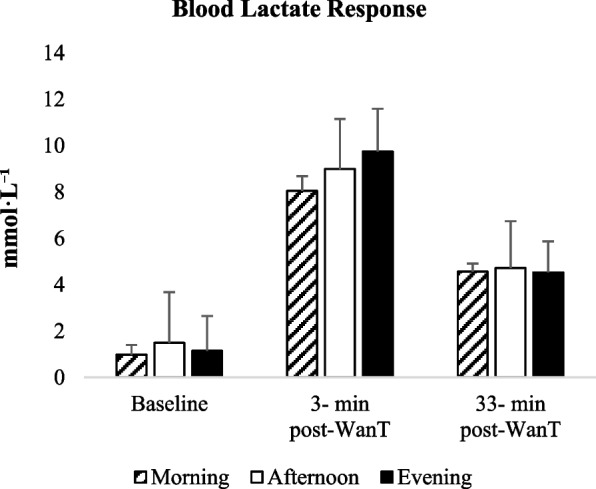
Blood lactate concentrations (mmol·L⁻^1^) measured at baseline, 3 min post-exercise, and 33 min post-exercise during morning, afternoon, and evening sessions. Error bars represent standard deviation

Blood lactate responses did not exhibit a significant time-of-day effect at either post-exercise measurement point (see Fig. 5). Analysis of blood lactate concentrations measured at the 3rd minute post-exercise revealed no statistically significant differences between morning, afternoon, and evening sessions, F(2, 40) = 3.169, p = 0.053, η^2^ₚ = 0.137. Bayesian analysis provided only anecdotal evidence for including the time-of-day factor (BF_incl = 2.179). Although lactate values tended to be higher in the afternoon and evening, these differences did not reach statistical significance; therefore, no post hoc comparisons were performed.

Similarly, blood lactate concentrations measured at the 33rd minute of recovery showed no significant effect of time of day, F(2, 40) = 0.193, p = 0.796, η^2^ₚ = 0.010. Bayesian analysis strongly supported the null model (BF_incl = 0.150), indicating the absence of a time-of-day effect. Accordingly, post hoc analyses were not conducted.

### Rating of Perceived Exertion (RPE)

RPE did not show a significant time-of-day effect across the testing sessions (see Table 3). Analysis revealed no statistically significant differences in RPE scores between morning, afternoon, and evening measurements, F(2, 40) = 0.680, p = 0.488, η^2^ₚ = 0.033. Bayesian analysis strongly supported the null hypothesis, providing no evidence for including the time factor in the model (BF_incl = 0.219). Accordingly, no post hoc comparisons were performed.

**Table 3:** Rating of Perceived Exertion (RPE)

TİME	Mean/Std	Rating of Perceived Exertion (RPE)				
		F	P	n2	BF10	BF ıncl
Morning Test	11.90 ± 2.64	0.680	0.488	0.033	1.000	0.219
Afternoon Test	11.76 ± 3.09					
Evening Test	11.23 ± 2.87					

## Discussion

This study investigated the effects of the Wingate Anaerobic Performance Test results—specifically, PPO, (W·kg⁻^1^), APO, (W·kg⁻^1^), and FI—along with (RPE) and blood lactate concentrations (measured at the 3rd minute peak and 33rd minute recovery) in trained women athletes who have consistently trained for the past year. The tests were conducted at three different times of day: morning (08:00–09:00 h), afternoon (13:00–14:00 h), and evening (18:00–19:00 h). The findings revealed significant differences in anaerobic performance based on the time of day, with evening performance being superior to morning performance (PPO, W·kg⁻^1^: 18.39%; APO, W·kg⁻^1^: 19.55%) and also better than afternoon performance (APO, W·kg⁻^1^: 8.06%). However, no similar effects were observed regarding the FI, RPE and blood lactate concentrations.

When examining the studies in the literature that investigate the effects of time of day on short-term maximum exercise performance, such as the Wingate test, several findings emerge that are both consistent and contradictory to the results of the current study. In the study [43] conducted the Wingate test on 10 elite-trained male taekwondo athletes during morning and evening periods. The study's findings indicated that there were no significant differences in PPO, APO and FI values based on the time of day. One factor contributing to this outcome is the participants' chronotype distribution heterogeneity. Although approximately 70% of the group exhibited an intermediate chronotype, the measurements were only taken during morning and evening hours, excluding the afternoon hours when these individuals' performances might have peaked. This limitation may have hindered the detection of a potential time-of-day effect. For instance [1], the Wingate test was administered to 36 football players with intermediate chronotypes at two different times of day—morning (08:00) and evening (17:00)—to examine performance variations related to time of day. The results revealed that both PPO and APO were significantly higher in the evening compared to the morning. However, the time of day had no significant effect on the FI. In another study [55], divided 24 untrained young male participants into three groups: morning training group (MTG), evening training group (ETG), and control group (CG), and examined the effects of a six-week strength training process on Wingate test performance depending on the time of day. According to the study's findings, in the tests performed before and after the training period, the PPO and APO values were significantly higher in the evening compared to the morning measurements. On the other hand, no significant difference was detected in the FI parameter depending on the time of day. Similarly, other studies reported substantial increases in PPO and APO outputs depending on the time of day, but no significant change was observed in the FI parameter [13, 18, 24]. These findings suggest that anaerobic power outputs may be affected by circadian rhythm, but parameters such as FI may be more complex and dependent on individual differences.

In contrast, some studies have reported significant increases in PPO, APO outputs, and FI values during the evening hours. These findings are inconsistent with the results of the present study, which did not identify a significant time-of-day effect regarding FI. This suggests that the influence of circadian rhythm on various components of anaerobic performance may differ across studies. For instance [36], found that PPO, APO, and FI data were significantly higher in the evening than in the morning during the Wingate test conducted with 20 physically active intermediate-type men in the morning and evening. In another study [26], reported that PPO, APO, and FI outputs were significantly elevated in the evening compared to the morning in the Wingate test administered to 15 trained young football players. Additionally, various studies employing different sample groups and methodological approaches have consistently shown that the PPO, APO, and FI parameters associated with Wingate test performance are significantly higher in the evening than in the morning [14, 17, 54]. One possible reason for the differences in the FI is the demographic and physiological diversity among the sample groups used in various studies. While many studies have focused on male athletes, the current research exclusively involved trained women participants. The physiological structures, hormonal cycles, and muscle fiber type distributions of women athletes may elicit different responses compared to their male counterparts, particularly regarding fatigue development and recovery processes [8, 60]. Furthermore, research has indicated that women exhibit greater fatigue resistance in locomotor muscles than men during single-limb and whole-body exercise modalities, attributed to morphological differences [4]. When considering all these factors collectively, the lack of alignment between the current study's findings and some existing literature regarding the FI may be related to these physiological differences specific to the participant group.

In anaerobic performance tests, not only power outputs but also biochemical indicators that reflect metabolic responses following exercise play a crucial role in performance evaluation. In this context, studies examining blood lactate levels have received limited attention and have produced contradictory findings regarding physiological responses based on the time of day. For instance, a study by [33] investigated the effects of supramaximal exercise at different times of the day on blood lactate responses. Fourteen male university students underwent the Wingate test three times: morning, afternoon, and evening. The study's findings indicated that PPO and APO in the afternoon were significantly higher than those recorded in the morning, demonstrating a notable time-of-day effect. Conversely, no significant differences were observed in blood lactate levels measured at the 3rd minute after the Wingate test, regardless of the time of day. Similarly [51], investigated the effect of time of day on blood lactate level by applying a 20-min maximal time trial protocol to 16 trained male mountain bikers during morning and evening sessions. The findings demonstrated that time of day did not significantly influence blood lactate levels. In particular, no statistically significant differences were observed in blood lactate concentrations measured at 1, 3, and 5 min post-exercise between the morning and evening. Similarly, there are studies in the literature reporting that the time of day does not have a significant effect on post-exercise blood lactate levels, and the findings obtained in this direction are consistent with the results of the current study [2, 3, 12, 16, 23, 25, 26, 37, 50]. In contrast to these results, some studies in the literature report significant differences in post-exercise blood lactate levels based on the time of day. For instance [28], investigated the effects of time of day on performance and metabolic response by administering the Yo-Yo intermittent recovery test to 15 elite male soccer players with moderate morning and intermediate chronotypes. The test was conducted in the morning (07:00–08:30) and evening (17:00–18:30), revealing significantly higher blood lactate levels and total distance covered during the evening hours. These findings suggest that higher evening lactate responses may be associated with improved performance outcomes. Additionally, other studies in the literature report significant variations in post-exercise blood lactate levels depending on the time of day, with various studies supporting these relevant findings [9, 19, 44, 47]. It is thought that the differences reported in the literature in post-exercise blood lactate levels based on the time of day may be influenced by numerous variables, including the type and duration of the exercise protocol, timing of measurement, environmental conditions, as well as the age, gender, training status, chronotype characteristics, and metabolic response differences among participants. In this context, the absence of time-of-day differences in post-exercise blood lactate levels observed in the present study suggests that diurnal variations in anaerobic power output do not necessarily translate into parallel changes in lactate accumulation or recovery. In particular, the impact of hormonal fluctuations associated with circadian rhythms on muscle metabolism and lactate clearance may contribute to inter-individual response differences, even within similar test protocols. A careful consideration of these variables is essential to explain why significant time-of-day effects are observed in some studies, while absent in others.

In interpreting the physiological responses to exercise, the RPE data, which reflects the individual's subjective experience, is considered an essential complementary element. In this context, the current study examined RPE values obtained at different times and found no significant changes based on the time of day. This finding indicates that perceived exertion may remain stable across the day, even when objective measures of anaerobic performance exhibit time-of-day–related variation. While some studies in this field reported significant variations related to the time of day [10, 27, 35, 37, 40], others did not observe any significant time of day effect under similar conditions [19, 29, 34, 41, 45, 47]. These contradictory findings in the literature indicate that a single variable cannot explain perceived exertion levels related to the time of day and that numerous individual and environmental factors may influence the data on this subject. Factors such as participants' psychophysiological alertness levels, sleep quality, motivational status, chronotype characteristics, and the duration and intensity of the applied test protocol may have direct or indirect effects on ratings of perceived exertion. Furthermore, the subjective nature of RPE as a measurement tool may increase inter-individual perception differences and limit the time sensitivity of the results. Therefore, carefully considering contextual factors when interpreting RPE data will ensure that the results are addressed more consistently and meaningfully.

Several methodological limitations should be considered when interpreting the findings of the present study. First, the study was conducted exclusively with trained women athletes, which may limit the generalizability of the results to other populations and necessitates consideration of sex-specific physiological characteristics. Additionally, although participants received standardized pre-testing instructions—including abstaining from physical activity, caffeine, and alcohol, as well as maintaining consistent dietary intake before each session—key circadian-related variables such as sleep duration and quality, meal timing, individual chronotype, and menstrual cycle phases were neither objectively monitored nor experimentally controlled. These factors may influence metabolic responses, fatigue development, and recovery kinetics, potentially contributing to inter-individual variability in blood lactate concentrations and performance outcomes. Furthermore, blood lactate measurements were obtained at discrete time points (baseline, 3 min post-exercise, and 33 min into recovery), which may not fully capture individual differences in peak lactate appearance or early post-exercise dynamics. Therefore, the present findings—particularly those related to the absence of time-of-day differences in blood lactate responses—should be interpreted with caution. Future studies incorporating objective monitoring of circadian-related variables, menstrual cycle control, and more frequent lactate sampling may provide a more comprehensive understanding of time-of-day–related physiological responses.

## Conclusion

This study investigated the effects of different times of day on anaerobic performance outputs, blood lactate responses, and the RPE in trained women. The findings revealed significant differences in PPO (W·kg⁻^1^) and APO (W·kg⁻^1^) outputs across time of day. Power outputs recorded in the evening were significantly higher than those measured in the morning. Additionally, measurements taken at noon yielded APO values that were considerably higher than those taken in the morning (W·kg⁻^1^). These results support the notion of increased performance levels throughout the day. On the other hand, no significant differences were observed in FI, RPE, or blood lactate levels across time of day. No statistically significant changes were detected in blood lactate levels measured at the 3rd minute following the Wingate test or in recovery measurements taken at the 33rd minute. This suggests that anaerobic power outputs are more sensitive to the time of day, while individual and environmental factors may have a greater influence on metabolic and subjective responses.

## Data Availability

All data supporting the findings of this study have been deposited in Figshare and are accessible via the following link: https://figshare.com/s/0a1cd23ad96abda92151?file=55994417
